# Preparation and Immobilization Mechanism of Red Mud/Steel Slag-Based Geopolymers for Solidifying/Stabilizing Pb-Contaminated Soil

**DOI:** 10.3390/ma17133353

**Published:** 2024-07-06

**Authors:** Xinyang Wang, Yongjie Xue

**Affiliations:** 1 Shandong Hi-Speed Engineering Test Co., Ltd., Jinan 250098, China; mogutoubisheng@163.com; 2 State Key Laboratory of Silicate Materials for Architectures, Wuhan University of Technology, Wuhan 430070, China

**Keywords:** red mud, geopolymer, Pb, soil, steel slag

## Abstract

Pb-contaminated soil poses serious hazards to humans and ecosystems and is in urgent need of remediation. However, the extensive use of traditional curing materials such as ordinary Portland cement (OPC) has negatively impacted global ecology and the climate, so there is a need to explore low-carbon and efficient green cementitious materials for the immobilization of Pb-contaminated soils. A red mud/steel slag-based (RM/SS) geopolymer was designed and the potential use of solidifying/stabilizing heavy metal Pb pollution was studied. The Box–Behnken design (BBD) model was used to design the response surface, and the optimal preparation conditions of RM/SS geopolymer (RSGP) were predicted by software of Design-Expert 8.0.6.1. The microstructure and phase composition of RSGP were studied by X-ray diffractometer, Fourier transform infrared spectrometer, scanning electron microscopy and X-ray photoelectron spectroscopy, and the immobilization mechanism of RSGP to Pb was revealed. The results showed that when the liquid–solid ratio is 0.76, the mass fraction of RM is 79.82% and the modulus of alkali activator is 1.21, the maximum unconfined compressive strength (UCS) of the solidified soil sample is 3.42 MPa and the immobilization efficiency of Pb is 71.95%. The main hydration products of RSGP are calcium aluminum silicate hydrate, calcium silicate hydrate and nekoite, which can fill the cracks in the soil, form dense structures and enhance the UCS of the solidified soil. Pb is mainly removed by lattice immobilization, that is, Pb participates in geopolymerization by replacing Na and Ca to form Si-O-Pb or Al-O-Pb. The remaining part of Pb is physically wrapped in geopolymer and forms Pb(OH)_2_ precipitate in a high-alkali environment.

## 1. Introduction

Over the past few decades, the country’s rapid economic development, the dramatic increase in industrial activity and the growing size of its cities have caused widespread contamination of land, posing a serious threat to humans and the environment [1]. The communique of the National Soil Pollution Survey issued by the Ministry of Environmental Protection and the Ministry of Land and Resources of China shows that 1.5% of soil is contaminated with the heavy metal lead nationwide [2]. The heavy metal Pb in soil can pass through the food chain and become enriched in edible crops, which not only poses a serious threat to agroecological sustainability and food security, but also causes human health problems [3]. Therefore, Pb-contaminated soil has to be remediated urgently.

Remediation of Pb-contaminated soils has always been a technical challenge, and commonly used remediation techniques include chemically-enhanced scrubbing, chemical adsorption/precipitation, electrokinetic extraction and phytoremediation [3]. Solidification/stabilization (S/S) is a low-cost, fast-acting soil remediation technology. Ordinary Portland cement (OPC) is widely used in S/S technology because of its low price and wide range of applications [4]. However, the production and manufacturing process of OPC emits a large amount of CO_2_. According to statistics, the cement industry accounts for 5% of global CO_2_ emissions [5]. The use of large quantities of OPC increases carbon dioxide emissions, reinforcing the greenhouse effect and negatively affecting ecology and the climate worldwide. It is, therefore, necessary to explore low-carbon and efficient green cementitious materials that can replace OPC for the S/S of Pb-contaminated soils.

Geopolymer is a potentially green and environmentally friendly gelling material with the ability to immobilize heavy metals. With very low CO_2_ emissions compared to OPC and excellent mechanical strength and durability, geopolymers are widely used in the building materials industry [6]. Geopolymers are prepared by activating silica- and aluminum-rich industrial solid wastes under alkaline conditions and have a zeolite-like structure, which can provide closed cage-like cavities to immobilize heavy metals [6], or even induce heavy metals to participate in geopolymerization for the purpose of heavy metal immobilization [7].

Red mud (RM) and steel slag (SS) are two common bulk industrial solid wastes in China which are widely used in the road building materials industry because of their large production volume and cheap and easy availability. In recent years, the use of red mud and steel slag to prepare geopolymers for use as S/S binders has also received increasing attention. Zhang et al. [8] used red mud and blast furnace slag to prepare geopolymers for the immobilization of the heavy metals Pb^2+^ and Cr^3+^, achieving immobilization rates of 91.46% and 95.86%, respectively. Wang et al. [9] used red mud, blast furnace slag and phosphogypsum to prepare cementitious materials for the treatment of multi-metal contaminated soils, and the compressive strength of the cured soil was 350 kPa, and the leaching concentration of heavy metals could reach the Chinese hazardous waste standard. Feng et al. [10] prepared a novel steel slag-based binder for curing/stabilizing soils at Pb, Cd and Zn do-not-let sites, and found that the binder could convert heavy metals from soluble to stabilizing fractions. Therefore, the use of RM and SS to prepare geopolymers to stabilize heavy metal Pb-contaminated soils is an effective and feasible approach to immobilizing heavy metals through hydration and geopolymerization reactions, thus, reducing the impact of contaminated soils on the environment.

Currently, there are few studies on the use of RM and SS preparations of geopolymers for immobilization of Pb-contaminated soil and the mechanism of immobilization of heavy metal Pb in soil by RM/SS geopolymers has not been investigated. Therefore, in this study, RM and SS were used to prepare soil contaminated by heavy metal Pb. The response surface method was used to optimize the preparation conditions, and the relationship between the liquid–solid ratio, mass fraction of RM and modulus of alkali activator and the unconfined compression strength of the cured soil and the immobilization efficiency of Pb was explored. The immobilization mechanism of Pb in soil by RM/SS-based geopolymers was studied by comparing the microstructure, mineral composition and bonding state of the geopolymers before and after adding heavy metal Pb.

## 2. Materials and Methods

### 2.1. Raw Materials

Soil used in this study was collected from a construction site of a motorway section in Shandong Province, China. The collected soil was naturally air-dried, crushed and passed through a 2 mm-mesh sieve for subsequent experiments and tests. The optimum moisture content and maximum dry density of soil was tested (JTC E51 2009) [11]. the liquid limit and plastic limit of the soil was tested by Digital Display Soil Liquid-Plastic Limit Combined Tester (Nanjing Ningxi Soil Instrument Co., LTD, GYS-2, Nanjing, China), and the plasticity index of the soil was calculated according to Equation (1) (JTG 3431-2020).

(1) $$I_{P}=w_{L}-w_{p}$$

where *I_p_* is plasticity index, *w_L_* (%) is liquid limit and *w_p_* (%) is plastic limit.

The results are listed in Table 1. Based on the results, it can be seen that the soil has a plasticity index of 10.0 and an optimum moisture content of 10.3%.

Pipette method (HJ 1068-2019) [12] were used to test the particle size distribution of soil, and the results are shown in Table 2. From Table 2, it is known that the particle size of the soil is mainly distributed in the range of 0.02–0.25 nm.

**Figure 1:**
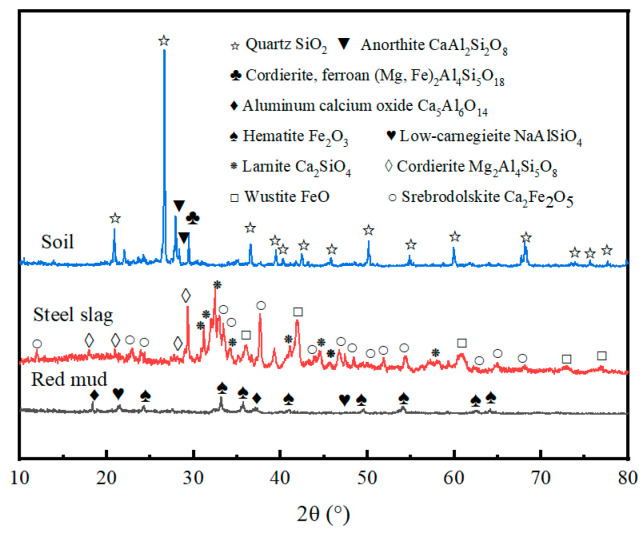
XRD of soil, RM and SS.

RM and SS used in the experiment were purchased from an industrial solid waste recycling plant in Shandong. Before experiment, planetary ball mill (MITR, YXQM-2L, Changsha, China) with a speed of 750 r/min was used to grind RM and SS. The ground material was screened with an 80-mesh screen and dried at 105 °C in drying ovens. The chemical composition and crystalline phase composition of the raw materials were examined using X-ray fluorescence (XRF, Rigaku, ZSX Primus III+, Tokyo, Japan) and X-ray diffractometer (XRD, Bruker, D8 Advance, Karlsruhe, Germany), the results of which are shown in Table 3 and Figure 1, respectively. The dominant crystalline phase of the soil is quartz (PDF#99-0088), with minor amounts of anorthite (PDF#41-1486) and cordierite ferroan (PDF#09-0472) also present. RM is mainly composed of Fe_2_O_3_, Al_2_O_3_, SiO_2_ and CaO. Therein, Al_2_O_3_ and SiO_2_ can provide Si and Al for the skeleton formation of geopolymer, and CaO can generate C-S-H gels or crystalline phases during geopolymerization, which provide strength to the geopolymer. The main crystalline phases of RM are hematite (PDF#33-0664), low-carnegieite (PDF#44-1496) and aluminum-calcium oxide (PDF# 03-0149). SS is mainly composed of CaO, Fe_2_O_3_ and SiO_2_, in addition to some MgO, Al_2_O_3_ and other oxides. The main crystalline phases of SS are cordierite (PDF#13-0294), larnite (PDF#33-0302), srebrodolskite (PDF#38-0408) and wustite (PDF#06-0615). Pb(NO_3_)_2_ and NaOH used in the experiment were purchased from Sinopharm Group Chemical reagent Co., LTD in Shanghai, China, and water glass was provided by Guangzhou Suixin Chemical Reagent Co., LTD in Guangzhou, China.

### 2.2. Preparation of Soil Samples

To ensure homogeneity and reproducibility of the soil used in the experiment, we used artificially prepared Pb-contaminated soil. Pb(NO_3_)_2_ (Sinopharm Group Chemical reagent Co., LTD, Shanghai, China) was used to prepare Pb-contaminated soil in this experiment because nitrate forms simpler compounds with the heavy metal Pb and has higher solubility [13]. With reference to the tertiary standard value (500 mg/kg) (GB 15618-1995) [14], a solution of heavy metal Pb at twice the standard value was added to the soil, stirred well, wrapped in plastic bags and aged for a fortnight in natural cool environment to simulate actual contaminated soil. The soil was dried in an oven at 105 °C before use.

**Figure 2:**
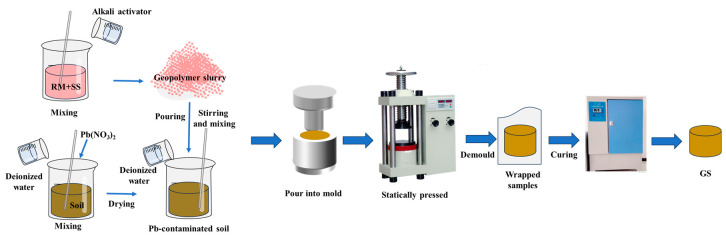
GS preparation process flow chart.

First, alkali activator of different modulus was designed using 8 mol/L NaOH solution and water glass of modulus 3.3. Then, according to the experimental design, different content levels of RM and SS were weighed and mixed well, and the alkali exciter was added and stirred fully to obtain the geopolymer slurry. Typically, geopolymer-based soil curing agents are dosed at 15 wt% [15], so, in this study, the dosage of geopolymer used to cure the soil was 15 wt%. The geopolymer slurry was poured into the dry soil, in accordance with the optimum water content of 10.3 wt%, adding the appropriate amount of deionized water and mixing thoroughly. After mixing, the mixture was poured into a circular mold with a diameter of 40 mm and statically pressed into shape using a universal pressure tester with load of 30 kN and kept for 2 min to obtain the geopolymer-soil sample (GS). The GS was encapsulated in a sealed bag and sent to a spray-conditioning room at 25 °C for 3d. GS preparation process flow chart is shown in Figure 2.

### 2.3. Testing Methods

In this study, the unconfined compressive strength (UCS) was carried out (JTC E51 2009) [11]. The formula for calculating the unconfined compressive strength is shown in Equation (2).

(2) $$R_{C}=\frac{F_{C}}{A}$$

where *R_C_* (MPa) is the compressive strength of geopolymer, *F_C_* (N) is the maximum pressure on the geopolymer and *A* (mm^2^) is area of the compressed part of the geopolymer, which was 1256 mm^2^ in this study.

After the UCS test, the crushed samples were collected, dried and ground through an 80-mesh sieve for the toxicity leaching test (HJ/T3 300 2007) [16]. The immobilization efficiency of Pb in geopolymer on Pb in soil was calculated according to Equation (3).

(3) $$\varphi =\frac{C_{0}-C}{C_{0}}\times 100\%$$

where φ (%) is the immobilization efficiency of Pb, C_0_ (mg/L) is the concentration of Pb leaching from contaminated soil and C (mg/L) is the leaching concentration of Pb in soil after the addition of geopolymers.

### 2.4. Optimization of Preparation Conditions

This experiment was carried out to optimize the preparation process of geopolymer by developing response surface methodology (RSM). The Box–Behnken design (BBD) model was adopted with the mass fraction of RM, liquid–solid ratio and modulus of alkali activator as independent variables, and UCS and immobilization efficiency of Pb as response indicators. Based on the results of the previous pre-experiment, the feasible ranges of the independent variables were screened and three-level experiments were designed, as shown in Table 4.

### 2.5. Characterization of Samples

RM/SS geopolymer (RSGP) was prepared according to the optimal preparation conditions. The material of SS and RM was weighed and mixed well with alkali exciter, then poured into the 2 mm × 2 mm × 2 mm mold, vibrated and pounded, wrapped with plastic film and sent to a spray curing room (25 °C) for 3d. Because of the complex composition of the contaminated soil [13], Pb(NO_3_)_2_ solution was mixed with RSGP slurry to obtain geopolymer containing Pb (RSGP-Pb), and the interaction mechanism between RSGP and Pb was investigated. After curing for 3d, RSGP and RSGP-Pb were crushed, and block samples of about 0.5–1 cm were collected to obtain information on the microstructure of RSGP and the elemental content on the surface of the material using scanning electron microscopy (SEM, Tescan, MIRA, Brno, Czech Republic). The samples were collected and passed through the 200-mesh sieve and the crystalline phase composition of the samples was determined by X-ray diffractometer (XRD, Bruker, D8 Advance, Karlsruhe, Germany), with a set scanning interval of 5° to 90° and a scanning step of 5°/min. The RSCP and RSGP-Pb were tested using Fourier transform infrared spectrometer (FTIR, Thermo Scientific, Nicolet iS20, Waltham, MA, USA) with wavenumber range of 400–4000 cm^−1^ to obtain information about the functional groups on the surface of the material. X-ray photoelectron spectroscopy (XPS, Thermo Scientific, ESCALAB 250Xi, Waltham, MA, USA) was used to probe the chemical state and molecular structure information of the elements on the surfaces of RSGP and RSGP-Pb, and all peaks were corrected according to C-C (284.8 eV).

## 3. Results and Discussion

This section is divided by subheadings. It should provide a concise and precise description of the experimental results and their interpretation, as well as the experimental conclusions that can be drawn.

### 3.1. Model Construction and Significance Analysis

The corresponding experimental design for the BBD model is shown in Table 5. After curing GS for 3d, an unconfined compressive strength test and the leaching experiment for Pb were carried out and the results are displayed in Table 5. The data were imported into software of Design-Expert 8.0.6.1 and the experimental results were fitted and analyzed using Quartic regression equations, the results of which are shown in Table 6.

As shown in Table 6, the F-value of the model is 21.28, with a *p*-value (0.0003) < 0.05, indicating that the model is significant [17]. The lack of fit F-value of 2.18 implies the lack of fit is not significant relative to the pure error. There is a 23.26% chance that a lack of fit F-value this large could occur due to noise. Meanwhile, the model fitting correlation coefficient (R^2^ = 0.9647) was in agreement with value R_adj_^2^ (0.9104). With the above results, the constructed model can explain the variation in unconfined compressive strength.

In order to verify the accuracy of the response surface regression model, a significance level of α = 0.05 was set and each term of the model was tested for significance using the F-distribution. When the *p*-value is less than 0.05, the model term is considered significant and can be included in the optimization analysis. Whereas, when the *p*-value is greater than 0.1, it indicates that the model term is not significant. In this case, A, B, C, BC, A^2^, B^2^ and C^2^ are significant model terms, while the AB and AC terms are insignificant and should be excluded from the optimization analysis. After excluding the non-significant terms, the optimized regression equation is expressed as

Y_1_ = 3.63 − 0.098 × A − 0.14 × B + 0.092 × C + 0.18 × B × C − 0.28 × A^2^ − 0.46 × B^2^ − 0.28 × C^2^ (4)

For Pb immobilization efficiency, according to Table 7, the model F-value is 114.20, with a *p*-value < 0.0001, indicating that the model is extremely significant. The *p*-value of the lack of fit was 2.67 and the F-value (0.1829) was greater than 0.05, indicating that the influence of the lack of fit is not significant. This result indicates that the degree of deviation of the predicted values of the model from the data obtained from the test is small, and the modeling is reasonable and reliable. The value of R^2^ (0.9932) for the model fit is close to R_adj_^2^ (0.9846), indicating a good model fit. The results described above validate the correctness of the model’s description of the mathematical relationship between the dependent variable and Pb immobilization efficiency. The regression equation was optimized by checking the significance of each term of the model using F-distribution. Its results show that only the term AB is not significant. Therefore, the optimized regression equation expression is as shown below:

Y_2_ = 70.90 + 0.71 × A − 0.55 × B − 0.67 × C + 1.83 × A × C + 5.70 × B × C − 3.48 × A^2^ − 4.52 × B^2^ − 3.81 × C^2^ (5)

### 3.2. Response Surface Interaction Analysis

**Figure 3:**
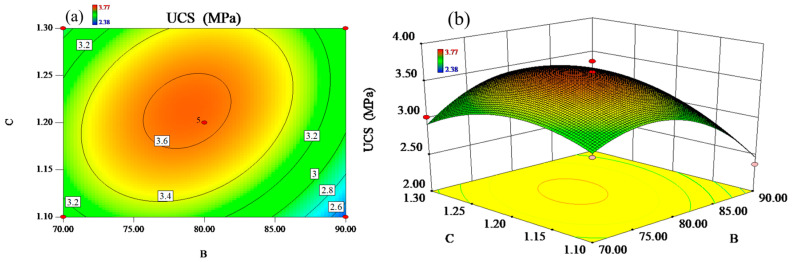
(**a**) The contours and (**b**) response surfaces of the interaction of B (the mass fraction of RM) and C (modulus of alkali activator) on UCS.

In order to analyze more intuitively the effect of the interaction of the mass fraction of RM and the modulus of alkali activator on the UCS, the data were subjected to response surface analysis based on the fitted equations to obtain 3D response surface maps and 2D contour maps under the limiting conditions. The results are shown in Figure 3.

Figure 3a demonstrates the interaction effect of the liquid–solid ratio and the mass fraction of RM on the UCS. As displayed in Figure 3a, the contour lines show a clear elliptical shape and are uniformly distributed on the horizontal and vertical coordinate axes with a comparable degree of denseness, indicating that the mass fraction of RM and modulus of alkali activator have a more obvious effect on each other in the limited range. Also, the slope of the response surface in Figure 3b is steeper, implying that the interaction of the mass fraction of RM and the modulus of alkali activator have a significant effect on the UCS.

When the liquid–solid ratio was fixed as 0.7, the UCS increased with the increase in RM mass fraction and decreased after reaching the peak value. The smaller change in UCS at lower RM content is due to the higher CaO content resulting from the higher steel slag content in the binary system. Higher CaO content, on the one hand, accelerates the hydration reaction and hardening rate of geopolymer to form more C-S-H and C-A-S-H gels [18]. On the other hand, higher CaO content promotes the condensation reaction of Si and Al to form a three-dimensional network structure, which enhances the UCS value of GS [19,20]. However, as the RM content increases, the SS percentage gradually decreases, as does the CaO content, and, thus, the UCS decreases. Keeping the mass fraction of RM constant, the value of UCS showed an overall trend of first increasing and then decreasing with the increasing modulus of alkali activator.

The alkali activator mainly provides a high-alkaline environment and sufficient Si source for the geopolymerization of RM and SS to increase the geopolymerization reaction rate [21]. When the alkali activator modulus is small, the amount of hydroxyl in the solution is higher. Higher alkali content leads to the creation of many cluster structures in the geopolymer during the early hydration stage, resulting in a lower UCS [22]. Whereas, as the alkali activator modulus increases, the alkali activator provides more silica monomers [22], which undergo a geopolymerization reaction with the dissolved Al from the raw material to form a rigid three-dimensional network structure. Under a suitable alkaline environment, the Si provided in the alkali active reacts with free Ca and Al ions to form C-S-H and C-A-S-H gels. With the enhancement of the maintenance time, Ca-containing phases are gradually formed to fill the soil pores and improve the densification of the GS, which in turn improves the value of the UCS [17]. However, when the alkali activator modulus is too large, the alkali content in the solution decreases, leading to insufficient hydration and geopolymerization reactions and a decrease in the reaction rate, therefore, resulting in a lower UCS.

**Figure 4:**
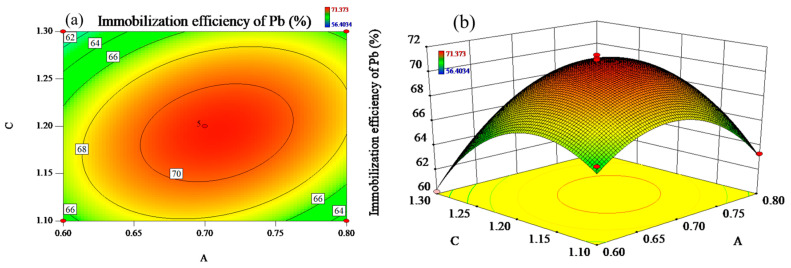
(**a**) The contours and (**b**) response surfaces of the interaction of A (liquid–solid ratio) and C (modulus of alkali activator) on immobilization efficiency of Pb.

Figure 4 reflect the effect of the interaction of liquid–solid ratio and modulus of alkali activator on the Pb immobilization efficiency at the constant mass fraction of RM (B = 80 wt%). As illustrated in Figure 4a, the contour lines show a distinct oval shape and are densely and uniformly distributed. Figure 4b shows that the slope of one response surface is steeper than Figure 3b, suggesting that the interaction of liquid–solid ratio and modulus of alkali activator has a more significant effect on the Pb immobilization efficiency.

When the alkali activator modulus was controlled to the fixed value, the immobilization efficiency of Pb increased with the liquid–solid ratio and reached the peak value of 71.37%. When the liquid–solid ratio exceeds 0.7, the immobilization efficiency of Pb decreases subsequently. Meanwhile, when the liquid–solid ratio is controlled to be constant, similar to the liquid–solid ratio, Pb immobilization efficiency presented the common tendency of first increasing and then decreasing as the modulus of alkali activator increased and reached the maximum value at the modulus of 1.2.

**Figure 5:**
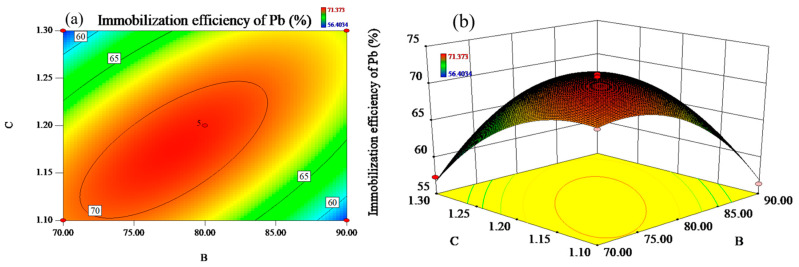
(**a**) The contours and (**b**) response surfaces of the interaction of B (the mass fraction of RM) and C (modulus of alkali activator) on immobilization efficiency of Pb.

With the liquid–solid ratio fixed at 0.7, Figure 5 exhibits the effect of the interaction of the mass fraction of RM and the modulus of alkali activator on the Pb immobilization efficiency. The contours are elliptical and more uniformly distributed, while the slope of the response surface is relatively steep, suggesting that the interaction of the mass fraction of RM and the modulus of alkali activator has a significant effect on the efficiency of Pb immobilization. As shown in Figure 5, the immobilization efficiency of Pb showed an increasing and then decreasing trend with the increase in the mass fraction of RM. Pb immobilization efficiency reached maximum value when the mass fraction of RM was 80%, which might be related to the change in Ca content in the system. When the mass fraction of RM was constant, Pb immobilization efficiency also showed an increasing and then decreasing trend with the increase in the modulus of alkali activator.

### 3.3. Model Validation

**Figure 6:**
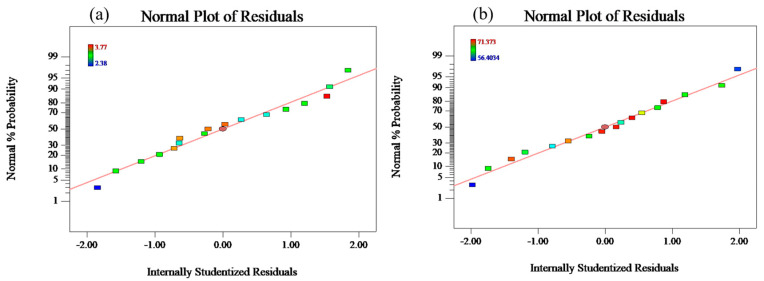
Residual plot (**a**) UCS and (**b**) immobilization efficiency of Pb.

In response surface design experiments, residual normal distribution plots are usually used to verify whether the model is consistent with the assumptions and to check whether there are abnormal experimental values. The result is shown in Figure 6a,b. As can be seen from Figure 6a,b, the data points are uniformly distributed on both sides of the straight line, indicating that the residuals of the regression model for the UCS and Pb immobilization efficiency of the GS are random, with few outliers, and are well-fitted to the actual situation.

**Figure 7:**
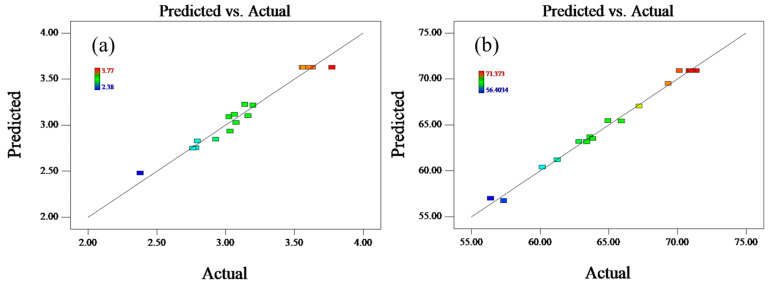
Predicted vs. actual experimental data of (**a**) UCS and (**b**) immobilization efficiency of Pb.

In addition, the veracity and validity of the regression model will also be judged by comparing the model predicted values with the experimental actual values. The relationship between the actual and predicted values of UCS and Pb immobilization efficiencies of GS is shown in Figure 7. The data points in the figure are uniformly distributed on both sides of the straight line and are very close to the line, implying that the actual values basically remain in agreement with the predicted values. Therefore, the optimum conditions obtained through the BBD model are reasonable.

### 3.4. Experimental Optimization and Validation Characterization of RSGP

Using the numerical module analysis of the Design-Expert software, UCS and Pb immobilization efficiencies were selected to be the maximum in the range of response values to obtain the optimum preparation conditions and model predictions, as shown in Table 8. According to Table 8, three sets of parallel samples were prepared and tested for UCS and Pb immobilization efficiencies, and mean values were taken to calculate the relative deviations. The results are shown in Table 8. The average values of UCS and Pb immobilization efficiency for the actual values were 3.42 MPa and 71.95%, respectively. The relative deviations of the actual and predicted values of UCS and Pb immobilization efficiencies were 1.72% and 1.31%, respectively, which were less than 5%, indicating that the model is scientifically sound, and the results obtained have a high degree of confidence.

### 3.5. Characterization of RSGP Immobilization Mechanism

**Figure 8:**
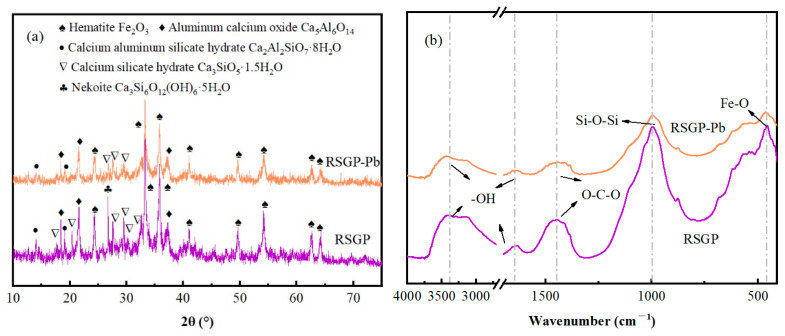
(**a**) XRD and (**b**) FTIR of RSGP and RSGP-Pb.

The XRD of RSGP is shown in Figure 8a. The presence of hematite and aluminum calcium oxide was observed in the diffraction pattern of RSGP. These phases were all from red mud, indicating that the 3-day curing was not sufficient for the raw materials to react adequately. In addition, there are still many new phases observed, such as calcium aluminum silicate hydrate (PDF#30-0227), calcium silicate hydrate (PDF#14-0035) and nekoite (PDF#31-0303). Therein, calcium aluminum silicate hydrate and calcium silicate hydrate are the products of the hydration reaction of raw materials. These crystalline phases can gradually fill the soil gaps during the maintenance process, forming a dense structure and enhancing the UCS of the cured soil [17]. At the same time, the hydration process of the raw material facilitates the entry of the heavy metal Pb from the soil into the structure of the geopolymer for effective solidification [17].

Figure 8b shows the FTIR of RSGP after curing for 3d. The peaks at around 3400 cm^−1^ and 1600 cm^−1^ are attributed to the stretching vibration of water molecules (-OH) in the RSGP [23]. The spectrum produces a weak peak belonging to O-C-O near 1440 cm^−1^, which may be the result of carbonate formation by atmospheric carbonation [23]. The peak located at 995 cm^−1^ is attributed to the asymmetric stretching vibration of Si-O-Si [24]. The peak at 460 cm^−1^ corresponds to the stretching vibration of Fe-O in hematite [25].

**Figure 9:**
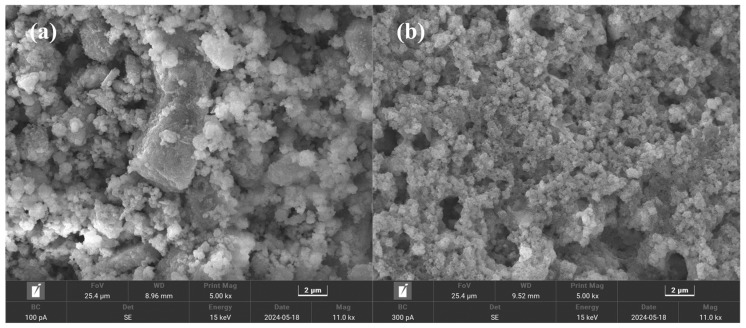
SEM of (**a**) RSGP and (**b**) RSGP-Pb.

The microscopic morphology of RSGP was observed by SEM. The results are shown in Figure 9a. As can be seen from Figure 9a, RSGP is highly hydrated and clearly exhibits a dense crystalline phase C-S-H structure, which is consistent with the XRD analysis. These crystalline phases are tightly bonded together, which not only improves the value of the UCS, but also provides more active sites for Pb immobilization.

### 3.6. Immobilization Mechanism

The immobilization mechanism of RSGP on Pb was analyzed using XRD, FTIR and SEM, and the result are displayed in Figure 8 and Figure 9. As exhibited in Figure 8a, compared to RSGP, part of the diffraction peaks of calcium aluminum silicate hydrate and calcium silicate hydrate in RSGP-Pb disappeared, suggesting that calcium aluminum silicate hydrate and calcium silicate hydrate may play a major role in reducing Pb leaching. The reason for this phenomenon is that Pb ions are sequestered in calcium aluminum silicate hydrate and calcium silicate hydrate by lattice immobilization, i.e., Pb ions are exchanged with Ca ions, and, thus, stably exist in calcium aluminum silicate hydrate and calcium silicate hydrate [26,27]. However, Pb-related diffraction peaks were not detected, which may be due to the short curing time (3d), Pb still existing in an amorphous state or the Pb-containing crystalline phases being masked by peaks in other complex crystalline phases. The intensity of the peaks in the RSGP-Pb spectrum is substantially weakened by the addition of the heavy metal Pb compared to RSGP, especially Si-O-Si, which suggests that the Pb undergoes ion exchange with RSGP [24]. Figure 9b demonstrates the microstructure of RSGP-Pb. The results show that the structure of RSGP-Pb becomes loose after the addition of heavy metal Pb. This may be due to the participation of Pb in the geopolymerization reaction, which generated a large amount of amorphous crystalline phase of Pb. These results echo the XRD.

**Figure 10:**
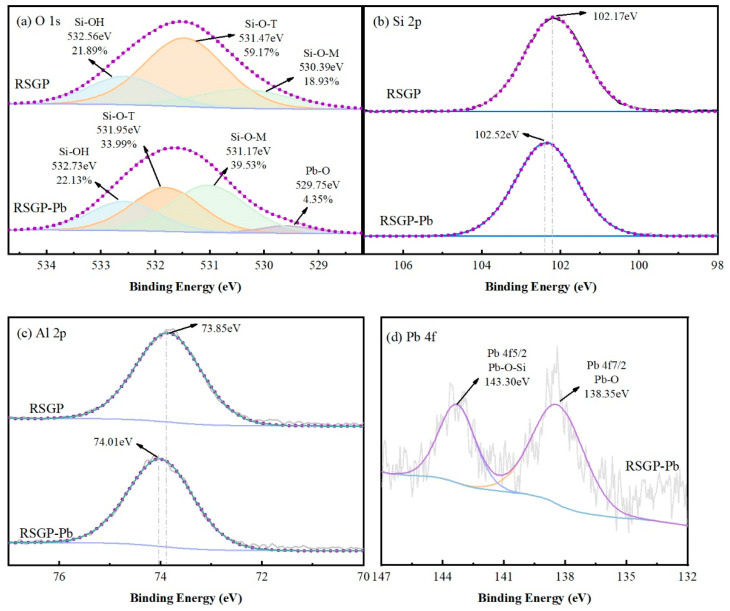
XPS of (**a**) O 1s, (**b**) Si 2p, (**c**) Al 2p and (**d**) Pb 4f of RSGP and RSGP-Pb.

The immobilization mechanism of RSGP on Pb was further verified using XPS, the results of which are shown in Figure 10. As shown in Figure 10a, the high-resolution O1s spectrum of RSGP was decomposed into three peaks located at 530.39 eV, 531.47 eV and 532.56 eV, which correspond to the Si-O-M (M = Ca or Na,), Si-O-T (T = Si or Al,) and Si-OH. The O1s binding energy of RSGP-Pb increased compared to RSGP and the percentage of Si-O-M increased from 18.93% to 39.53%, while the percentage of Si-O-T decreased from 59.17% to 33.99%, indicating that Pb is involved in geopolymerisation to produce Si-O-Pb. It has been shown that Pb participates in geopolymerisation in Si-rich high-alkaline environments, replacing some Ca and Na ions to form mono- or multinuclear ligands Si-O-Pb or Al-O-Pb. It was shown that Pb would participate in geopolymerisation and exchange with Ca and Na ions to form the mono- or polynuclear ligands Si-O-Pb or Al-O-Pb, immobilized within the geopolymer in a high-alkaline environment rich in Si [28,29]. In addition, the peak at 529.75 eV in RSGP-Pb corresponds to Pb-O [30], suggesting that part of the Pb is physically wrapped in the geopolymer and forms Pb(OH)_2_ in a highly alkaline environment [1]. Figure 10b,c show the high-resolution Si2p, Al2p spectra of RSGP and RSGP-Pb. The results show that the binding energies are all shifted to a higher direction after the addition of heavy metals, which also further confirms the involvement of Pb in the geopolymerization reaction to produce Si-O-Pb and Al-O-Pb [28]. Pb4f (Figure 10d) demonstrates similar results. The high-resolution Pb4f spectra show two peaks, Pb 4f7/2 and Pb 4f5/2, located at 138.35 eV and 143.30 eV. Two peaks in the high-resolution Pb4f spectrum are located at 138.35 eV and 143.30 eV and are attributed to Pb 4f7/2 and Pb 4f5/2, respectively. The peak of Pb4f7/2 at 138.35 eV is attributed to Pb(OH)_2_ [31], while the peak at 143.30 eV is consistent with the literature value of Si-O-Pb [32,33]. Considering all the above results, Pb is mainly immobilized in geopolymer by lattice immobilization through exchange with Ca, Na and other alkali metal ions, and complexes such as Si-O-Pb/Al-O-Pb are generated. On the other hand, part of the Pb is immobilized in RSGP by physical wrapped and generates Pb(OH)_2_ under a high-alkaline environment.

## 4. Conclusions

In this study, RM and SS were used as raw materials to prepare RSGP for solidification/stabilization of Pb-contaminated soil. The BBD model was used to design the response surface, and Design-Expert software was used to analyze the significance of regression equations of UCS and Pb immobilization efficiency. The results showed that the interaction of the RM mass fraction and the modulus of alkali activator had significant effects on UCS and Pb immobilization efficiency, while the liquid–solid ratio and modulus of alkali activator also had significant effects on the immobilization efficiency of Pb. The optimum preparation conditions of RSGP were as follows: 0.76 of liquid–solid ratio, 79.82% of RM mass fraction and 1.21 of alkali activator modulus. At this time, the UCS value of the solidified soil is 3.42 MPa, and the immobilization efficiency of Pb is 71.95%. XRD, FTIR, SEM and XPS were used to reveal the immobilization mechanism of Pb by RSGP. The results show that the main phases of RSGP are calcium aluminum silicate hydrate, calcium silicate hydrate and nekoite, which can enhance the UCS of solidified soil by filling the soil pores and improving the soil density. Pb in soil is mainly immobilized in RSGP through lattice immobilization, that is, Pb participates in the geopolymerization to generates Al-O-Pb and Si-O-Pb ligands. The remaining Pb was immobilized by physical wrapping and precipitated by Pb(OH)_2_ in a high-alkali environment.

## Figures and Tables

**Table 1:** The physical properties of soil.

Liquid Limit/%	Plastic Limit/%	Plasticity Index	Maximum Dry Density/g∙cm^−3^	Optimum Moisture Content/%
30.0	20.0	10.00	1.57	10.3

**Table 2:** Particle size distribution of soil.

Particle Size (mm)	Proportion (%)
<0.002	0
0.002–0.02	10.03
0.02–0.05	35.13
0.05–0.25	54.71
>0.25	0.13

**Table 3:** Chemical composition of the raw materials.

Chemical Composition	Mass Fraction/%		
	Red Mud (RM)	Steel Slag (SS)	Soil
Na_2_O	5.836	0.164	2.298
MgO	0.489	5.555	2.408
Al_2_O_3_	19.251	4.053	13.054
SiO_2_	11.367	16.345	56.416
K_2_O	0.349	0.037	4.428
CaO	9.942	40.188	11.636
Fe_2_O_3_	45.806	23.950	2.298

**Table 4:** The factors and levels of BBD model design.

Factors	The Minimum	The Maximum
A: Liquid–solid ratio	0.6	0.7
B: The mass fraction of RM (wt%)	70	90
C: Modulus of alkali activator	1.1	1.3

**Table 5:** Experimental scheme and the corresponding results.

Test Number	Proportion (%)			UCS (MPa)	Immobilization Efficiency of Pb (%)
	A	B	C	Y_1_	Y_2_
GS-1	0.7	90	1.1	2.38	50.33
GS-2	0.7	80	1.2	3.29	72.99
GS-3	0.6	90	1.2	2.79	61.26
GS-4	0.6	80	1.1	3.34	68.36
GS-5	0.7	80	1.2	3.91	72.59
GS-6	0.6	70	1.2	3.20	65.26
GS-7	0.8	70	1.2	2.79	63.63
GS-8	0.7	80	1.2	3.38	69.90
GS-9	0.7	90	1.3	3.08	67.22
GS-10	0.7	70	1.1	3.07	67.50
GS-11	0.6	80	1.3	3.14	60.17
GS-12	0.8	90	1.2	2.76	63.86
GS-13	0.7	80	1.2	3.77	70.15
GS-14	0.7	80	1.2	3.48	75.37
GS-15	0.7	70	1.3	3.03	59.78
GS-16	0.8	80	1.1	2.93	63.40

**Table 6:** Analysis of variance for regression models of unconfined compressive strength.

Source	F Value	*p* Value	
Model	21.28	0.0003	significant
A	6.68	0.0363	
B	12.95	0.0087	
C	5.95	0.0448	
AB	3.20	0.1166	
AC	0.31	0.5934	
BC	11.64	0.0113	
A^2^	29.49	0.0010	
B^2^	77.73	<0.0001	
C^2^	28.87	0.0010	
Lack of Fit	2.18	0.2326	not significant
$R^{2}$ = 0.9647		${R^{2}}_{Adj}$ = 0.9194	

**Table 7:** Analysis of variance for regression models of immobilization efficiency of Pb.

Source	F Value	*p* Value	
Model	114.38	<0.0001	significant
A	10.95	0.0129	
B	6.64	0.0367	
C	9.94	0.0161	
AB	2.22	0.1799	
AC	36.60	0.0005	
BC	356.59	<0.0001	
A^2^	140.23	<0.0001	
B^2^	236.04	<0.0001	
C^2^	167.36	<0.0001	
Lack of Fit	2.67	0.1829	not significant
$R^{2}$ = 0.9932		${R^{2}}_{Adj}$ = 0.9846	

**Table 8:** Optimal solution under comprehensive conditions generated by software and actual value.

Factor	A	B	C	Y_1_ (MPa)	Y_2_ (%)
Predicted	0.76	79.82	1.21	3.48	70.09
Actual				3.42	71.95
Relative deviation (%)	-	-	-	1.72	2.65

## Data Availability

The data presented in this study are available on request from the corresponding author. The data are not publicly available due to privacy.
